# A pilot study into the effects of music therapy on different areas of the brain of individuals with unresponsive wakefulness syndrome

**DOI:** 10.3389/fnins.2015.00291

**Published:** 2015-08-21

**Authors:** Nikolaus Steinhoff, Astrid M. Heine, Julia Vogl, Konrad Weiss, Asita Aschraf, Paul Hajek, Peter Schnider, Gerhard Tucek

**Affiliations:** ^1^OptimaMed Neurological RehabilitationKittsee, Austria; ^2^Department of Music Therapy, IMC University of Applied SciencesKrems, Austria; ^3^Department of Social and Cultural Anthropology, University of ViennaVienna, Austria; ^4^Department of Nuclear Medicine, Regional Hospital Wiener NeustadtWiener Neustadt, Austria; ^5^Department of Neurology, Regional Hospital HocheggGrimmenstein, Austria

**Keywords:** positron emission tomography (PET), music therapy, human brain, brain areas, activity alteration

## Abstract

The global cerebral network allows music “ to do to us what it does.” While the same music can cause different emotions, the basic emotion of happy and sad songs can, nevertheless, be understood by most people. Consequently, the individual experience of music and its common effect on the human brain is a challenging subject for research. Various activities such as hearing, processing, and performing music provide us with different pictures of cerebral centers in PET. In comparison to these simple acts of experiencing music, the interaction and the therapeutic relationship between the patient and the therapist in Music Therapy (MT) provide us with an additional element in need of investigation. In the course of a pilot study, these problems were approached and reduced to the simple observation of pattern alteration in the brains of four individuals with Unresponsive Wakefulness Syndrome (UWS) during MT. Each patient had three PET investigations: (i) during a resting state, (ii) during the first exposure to MT, and (iii) during the last exposure to MT. Two patients in the MT group received MT for 5 weeks between the 2nd and the 3rd PET (three times a week), while two other patients in the control group had no MT in between. Tracer uptake was measured in the frontal, hippocampal, and cerebellar region of the brain. With certain differences in these three observed brain areas, the tracer uptake in the MT group was higher (34%) than in the control group after 5 weeks. The preliminary results suggest that MT activates the three brain regions described above. In this article, we present our approach to the neuroscience of MT and discuss the impact of our hypothesis on music therapy practice, neurological rehabilitation of individuals in UWS and additional neuroscientific research.

## Introduction

During the 1980s and 1990s neuroscientific research predominantly used electroencephalography (EEG) to show music related activities in the brain (Pape, 2005). Today, 30 years later, more elaborate methods of investigation offer the opportunity to show cerebral processes related to music. Functional and structural changes are shown quite clearly using single or combined measurement techniques. Magnetic and functional magnetic resonance tomography (MRT, fMRT) brain mapping, positron emission tomography (PET) as well as magnetic encephalography (MEG) and other techniques are used to explore focal brain activities. These studies developed the evidence base for understanding how listening to music is a complex process that involves multiple brain regions. Besides the auditory cortex, music increases activity in frontal, temporal, parietal and subcortical regions (Koelsch, 2009; Altenmüller and Schlaug, 2015; Brown et al., 2015). Thus, music has a wide range of effects on emotion (Blood and Zatorre, 2001; Boso et al., 2006; Koelsch, 2006, 2009, 2015; Koelsch and Jentschke, 2010; Pereira et al., 2011; Vuilleumier and Trost, 2015), cognitive functions such as attention and memory (Särkämö et al., 2008; Baird and Samson, 2015; Castro et al., 2015), motor functions (Limb, 2006; Koelsch, 2009; Levitin and Tirovolas, 2009; Schaefer and Overy, 2015) and mood (Särkämö et al., 2008; Radstaak et al., 2014; Zatorre, 2015).

Due to the influence on the brain, listening to music and music therapy are often used in neurological rehabilitation of disorders of consciousness (Gustorff and Hannich, 2000; O'Kelly et al., 2013; Magee et al., 2014; Verger et al., 2014; Magee and O'Kelly, 2015). Unresponsive Wakefulness Syndrome (UWS) belongs to the disorders of consciousness and is one of the most severe neurological impairments. The damage in several brain regions leads to an inability to respond to the environment even though patients show clear signs of wakefulness (Adams et al., 2001; Gosseries et al., 2011). As a consequence, the severity of UWS manifests itself to those interacting with the patient in a sudden impossibility to communicate via the usual means. While most professionals try to support the detection and recovery of functional communication, music therapy additionally tries to find new ways of connecting and communicating within the framework of the patient's capabilities. Music therapy has been used to support the neural and behavioral rehabilitation of individuals with UWS for more than 20 years.

An increase in music therapy research in this field points to its importance (Gustorff and Hannich, 2000; O'Kelly et al., 2013; Magee et al., 2014; Magee and O'Kelly, 2015). Combined with research on the neurological impact of music, music therapy research leads to a better understanding of its benefits for patients with brain damage. Still, evidence of music therapy's impact on the neurological rehabilitation of individuals with UWS is rare. To improve our understanding of the impact of music therapy on the neurological rehabilitation and its neural processing, we propose to take a closer look into the brain during music therapy as a complex process.

Understanding music as a language that transports its own distinct neuropsychological and emotional codes (Spreckelmeyer et al., 2013), we follow the hypothesis that enhanced activity and functional augmentation in the cerebral regions for emotion, learning, motion planning, and cognition can be expected after music therapy and shown by PET. Furthermore, from a music therapy perspective, our hypothesis is that individual, live music therapy in a setting of a therapeutic relationship promotes the neurological rehabilitation of individuals with UWS and boosts their brain activity. Our approach may be considered as part of a developing approach exploring brain activation by music therapy, but with a particular focus on an individualized and open investigation format.

Our aim is to trigger new investigations as a dialogue between music therapy and neuroscience in an effort to heighten our understanding of the function of music therapy, its way of activating the brain and its implementation in neuro-rehabilitation. These investigations could also help improve the individual approach to each patient.

## Theoretical framework

What is the meaning of music therapy in its “whole complexity”? In our understanding, the complex effect of music therapy on the neuro-rehabilitation of individuals with UWS can be summarized in three aspects: the musical stimulus, the therapeutic relationship and the emotional exchange between the patient and therapist. To investigate the effect of music therapy, all three aspects need to be considered, not just the musical stimulus. For a better understanding of our hypothesis, the concept of music therapy as it is applied in Krems (Tucek, 2014; Tucek et al., 2014) needs a further explanation.

Music itself, as described in the introduction, has an impact on the human body, including brain, emotion, and movement, and so lends itself as an appropriate therapeutic medium in this and other fields. Even though studies show general neurological effects of music as a stimulus, the inter- and intra-individual meaning of this stimulus is different. Various aspects of listening to or performing music, such as personal preference, experience and the current mood are responsible for the formation of the personal meaning of music. Theories of embodiment (Csordas, 2002; Storch and Tschacher, 2014), emerging from anthropological studies, describe the engagement of culture and individuals through sensual perception and experience. Therefore, the meaning of music in therapy develops within the therapeutic session as a specific tool of communication between the patient and the therapist. To paraphrase Simon Rattle (2004), “music is not just what it is, but is that what it means to the people.” To perceive and respond to the personal meaning and individual reactions of patients, the therapist empathically observes the patient and constantly adapts the music and the whole interaction to the reactions of the patient (Eisenberger et al., 2003). This leads to a constant exchange between the patient and the therapist that forms the therapeutic process as well as shapes brain activity.

The foundation of this interaction is the therapeutic relationship. From early childhood, experiences of bonding and attachment enhance the growth and connectivity in the neural network (Schore, 1994), whereas social isolation increases the risk for morbidity and mortality (Cacioppo and Hawkley, 2003) and the potential for aggression (Eisenberger et al., 2003). Thus, interpersonal relationships are a basic need (Insel, 2001; Cozolino, 2006). Gustorff and Hannich (2000) emphasize that every living individual has the need and ability for perception and interpersonal communication. Although we do not know how patients with UWS perceive their environment, it is important to see them from a holistic perspective as social individuals. The therapeutic relationship has to be initiated and maintained actively in every session. Within the therapeutic relationship, we try to connect with the patients by observing their reactions to the performed music and by considering even the smallest physiological changes. Live music therapy can address the individual needs of patients and offer adjusted stimuli for the support of rehabilitation. We therefore propose that the experience of a therapeutic relationship within music therapy also promotes the connectivity in the neural networks in these patients.

Studies found that patients with UWS show emotional processing of auditory and visual information (Coleman et al., 2009; Yu et al., 2013). Music itself can evoke emotions (Koelsch, 2015). Additionally, emotional auditory stimuli, like listening to one's name or the mother's voice, activate anterior and posterior midline cortex in patients with UWS (Laureys et al., 2004; Demertzi et al., 2010). Emotion is a key component of how we experience our environment (Sharon et al., 2013). Emotional stimuli receive privileged access to attention and awareness, and thus are more likely to capture one's attention (Vuilleumier, 2005; Phelps, 2006). In particular autobiographic memories lead to emotional responses and involve widespread functions of the brain (Svoboda et al., 2006; Cabeza and St Jacques, 2007; Piolino et al., 2009). Music therapy uses this knowledge by applying familiar songs, singing names of individuals and using entrained music in therapy to reach the patients more directly and to promote reactions suggestive of awareness (Magee and O'Kelly, 2015).

A study on sensory stimulation revealed that, by inviting responses, we could pass from stimulation (which promotes arousal and attention) to rehabilitation (which promotes and reinforces behavioral responses) (Abbate et al., 2014). This statement supports our hypothesis that by combining musical stimuli, the therapeutic relationship and emotional approach, individual live music therapy encourages multi-sensory, behavioral and physical responses. These, in turn, promote the rehabilitation of individuals with UWS.

Until now, research on the neural effect of music therapy was limited to the observation of musical stimuli in the brain. To strengthen our understanding of the effect of music therapy in its complexity and to pretest our hypothesis, we started the first of a series of investigations of individual live music therapy. While the original research results will be published at a later date, part of the pilot study is presented in this article to describe our approach.

Even though inter-individual brain activity of the patients differs due to different levels of cerebral lesions, we expected to see functional and structural augmentation in the cerebral regions for emotion, learning, motion planning, and cognition (Schlaug et al., 2005; Hyde et al., 2009). In our pilot study, only patients with hypoxic brain injury following cardiopulmonary resuscitation (CPR) in UWS were chosen, where a more homogenous affection of the brain could be expected. While traumatic brain injury leads to heterogeneous regions of cortical damages in the brain with patterns of several foci, non-traumatic causes show an impact of thalamic and cortical functions due to hypoxic nerve cell lesions (Markl et al., 2013). Consequently, the fronto-temporo-parietal network also shows a decrease of activity in patients with UWS (Jennett, 2002; Laureys et al., 2004; Demertzi et al., 2010, 2013; Laureys and Schiff, 2012). However, for the first pilot study, we reduced our focus by limiting the examination to those three brain areas that are thought to be crucial to the success of music therapy and cognitive functions. Those are the frontal regions, the hippocampus and the cerebellum.

Following our hypothesis, the aim of this pilot study was to examine whether differences can be detected in the brain between individuals after hypoxic brain lesions who received music therapy and individuals with no music therapy in neurological rehabilitation.

## Material and methods

### Study organization

The pilot study was conducted at the IMC University of Applied Sciences Krems, under the direction of the corresponding author. The practical work with the patients was carried out at the Intermediate Care Unit (IMCU), specialized in rehabilitation of patients with disorders of consciousness at the Provincial Hospital of Hochegg, Austria. Ethical approval was given by the official Ethics Committee of Lower Austria. The study was financially supported by the Lower Austrian Health and Social Fund (NÖGUS) and Lower Austrian Provincial Hospital Holding (Landeskrankenhaus Holding). However, the sponsors had no role in study design, data collection, analysis, and interpretation.

### Participants

In this pilot study, we included patients with UWS after CPR who stayed at the IMCU. Patients were diagnosed before uptake at the IMCU and the UWS was confirmed after uptake following the common rules of diagnosis (Adams et al., 2001). The participants' legal representatives gave their written consent after a personal elucidation. Patients were randomly enrolled either into the music therapy group or the control group by drawing lots. For the first evaluation of our hypothesis, we examined four participants, two in the music therapy group and two in the control group.

### Methods

To show the activity of the brain during and after music therapy, we used PET investigations (Siemens Biograph 16 HiRez PET-CT Scan). PET is still the only method to study the relation between cognitive processes and neurotransmission by showing radiation of nuclear medical tracers in active brain areas (Pape, 2005; Akanuma et al., 2015).

Patients in both groups had three PET scans within 6 weeks: the first one (week 1) is a standard PET scan by the hospital in a resting state, without any stimulation. The second (week 2) and third (week 6) are with individual, live music therapy right before the PET scan and during the tracer application. The participants were transported from the hospital bed to the nuclear medical investigation at the Central Radiological Institute in Wiener Neustadt and received an intravenous ^18^F-FDG tracer application (230 MBq) during music therapy or a resting state in the PET room. By measuring ^18^F-FDG tracer uptake in the brain PET shows the activity of brain regions. The advantage of this form of investigation is that the tracer is applied during music therapy or resting state, and we can see which brain regions are active and compare the results of different situations and times.

Patients in the music therapy group received live and individual music therapy for 5 weeks between the 2nd and the 3rd PET scan, three times a week. The sessions were conducted by a trained music therapist using various instruments and the therapist's voice. The approach to the patients in the therapy sessions coheres with the theoretical frame described above. A key element of the therapeutic work was the attunement to the patient. The therapies started with an initial touch on the arm or shoulder and humming, singing, or playing in the rhythm of breath. The manner of breath allows for the interpretation of the patient's current constitution to which the therapy is adapted. Autobiographical information, such as favorite songs or artists, were involved in the therapy as well as singing the patient's name. The therapist carefully observed the patient the entire time, including his physical (e.g., tonicity, facial expression, eyes) and physiological (e.g., breath, heart rate, oxygen saturation) actions and reactions, and adjusted to these. For example to support relaxation the therapist played improvisations entrained to the rhythm of the patient's respiration, or in order to help the patient to relive tension the therapist enhanced the amount of smooth tactile contact. To invite reactions and to avoid excessive demand, music and speech were provided in a basal, slow and adjusted manner and filled with pauses. The average therapy duration lasted for 27 min. All sessions were recorded on video and documented in protocols for further analysis.

Patients in the control group had no music therapy during those 5 weeks. However, all participants received standard care (physical, occupational and speech therapy, neuropsychological treatment), as the pilot study took place at the IMCU Hochegg, specializing in neurological rehabilitation of individuals with UWS (Table 1).

**Table 1:** **Course of the study**.

	**Music therapy group (*n* = 2)**	**Control group (*n* = 2)**
Week 1	PET 1 (Rest)	PET 1 (Rest)
Week 2	PET 2 (Music therapy)	PET 2 (Music therapy)
Week 2–6	5 weeks standard care + music therapy (3 per week)	5 weeks standard care
Week 6	PET 3 (Music therapy)	PET 3 (Music therapy)

Three brain areas were analyzed in this pilot study, namely the frontal areas, the hippocampus and the cerebellum.

The frontal regions are known for processing cognitive and motor functions. For example, frontal premotor areas are involved in the perception and production of rhythm (Limb, 2006; Levitin and Tirovolas, 2009), while other frontal regions are responsible for cognitive tasks, impulsion, memory, and social functions (Trepel, 2008).

The hippocampus is a part of the limbic system, hence is involved in emotional processes, social bonding, and relationships (Koelsch, 2012). Therefore, it is hypothesized that therapeutic relationship may have an influence on the hippocampus. Its activity increases while listening to music that is associated with positive emotions (Brown et al., 2004; Koelsch, 2009; Levitin and Tirovolas, 2009). Additionally, it plays a crucial part in learning, memory, spatial orientation and the processing of sensory information (Blood and Zatorre, 2001; Brown et al., 2004; Eldar et al., 2007; Trepel, 2008; Levitin and Tirovolas, 2009; Koelsch, 2012).

The cerebellum is involved in several motor functions, such as posture, tonicity, and arbitrary movement (Trepel, 2008). Due to the connection to the limbic system it is also involved in cognitive and emotional processes. In musical tasks it is responsible for the perception and production of rhythm as well as emotional reactions to music (Blood and Zatorre, 2001; Limb, 2006; Levitin and Tirovolas, 2009; Trost et al., 2012; Akanuma et al., 2015).

This pilot study was based on the hypothesis that the brain is activated by individual music therapy. We assume that PET can be used to show that music therapy reliably activates the human brain and enhances neurological rehabilitation. However, our aim was not to prove our hypothesis, but observe the brain of individuals with UWS during music therapy and develop our understanding of this in the context of a neuroanthropological approach.

## Results

Quantitative data of uptake values were generated automatically using the Syngo Scenium Ver.1.2.0.13 Siemens Medical Solutions software. To avoid misinterpretation caused by metabolic variations, all results were adjusted to the uptake values of a reference region (calvaria). For further analysis the differences between the three PET scans were calculated for each patient individually (numerical value and percentage calculation) and then compared to each other. As the numerical values of the differences vary considerably due to the severity of the patients' brain lesions, the changes in the uptake values are presented in percentages. This allows a better comparison of the results in the two groups.

**Figure 1:**
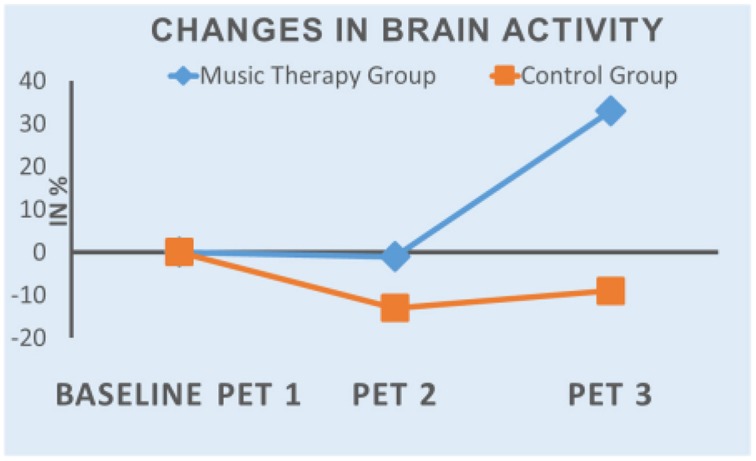
**Mean values of the changes in brain activity in the course of the study**.

The results of the first evaluation show an increase in tracer uptake in PET 3 in all three areas in music therapy patients, while it decreased in the control group patients. In both groups tracer uptake was lower in PET 2 than in PET 1 (mean value: MT-Group: −1%; CG: −12%). Figure 1 shows the mean values of the changes in the course of the study.

**Figure 2:**
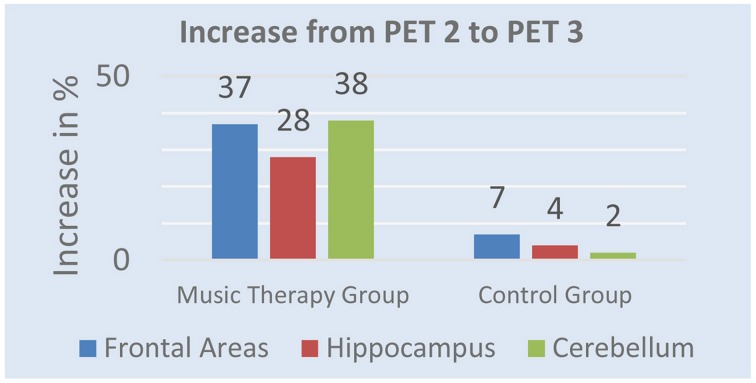
**Mean values of the changes from PET 2 to PET 3**.

After 5 weeks of music therapy tracer uptake in PET 3 increased by 37% in frontal regions, 28% in hippocampus, and 38% in cerebellum in the music therapy group. The control group shows different results. While activity increased in PET 3 by 7% in frontal areas, 4% in hippocampus and 3% in cerebellum, tracer uptake was still lower than in PET 1. Figure 2 shows the mean value of changes from PET 2 to PET 3.

The goal of the investigation was not to describe different states of consciousness or the awakening after music therapy but to show changes in brain activity before, during and after the therapy. This was documented clearly through simple PET investigation. However, we did not conduct further statistical analyses in this pilot study due to the small number of participants.

## Interpretation

Considering the low number of subjects, we want to handle the interpretation with care. However, there is a considerable difference between the two groups, which supports our hypothesis. The increase of tracer uptake can be interpreted as an increase in brain activity. Patients in the music therapy group show a higher brain activity than control group patients. However, we have to take into account that PET 3 is also a scan of music therapy as a stimulus. We cannot yet explain the decrease of tracer uptake in PET 2, as patients received the first music therapy during this situation and four cases provide insufficient data for interpretation.

This pilot study represents a first step in a series of investigations as a dialogue between music therapy and neuroscience. It shows that these research methods may open the way to getting more definite results on the effect of music therapy on the neuro-rehabilitation of individuals with UWS. While this pilot study focused on the activity in targeted areas of the brain, further research will provide more room for interpretation of the neurological rehabilitation. Additionally, more patients and statistical analysis of the PET results may help clarify our results.

## Discussion

This pilot study was a primary step into the very complex field of music therapy in the neuro-rehabilitation of patients with UWS. Examining four patients was the first attempt to evaluate whether our hypothesis can be tested with the chosen methods. Further research is currently in progress and the following steps are planned. In light of the complexity of music therapy as discussed previously, the focus on three brain areas is a limiting factor. Nevertheless, it was a stepping stone for developing research methods under almost-bedside conditions in order to bridge the gap between research and practice.

During rehabilitation of individuals with UWS, neurologists and music therapists have a long history of interdisciplinary cooperation. Particularly when working with patients who cannot communicate what they experience, it is important to find indications from the effect of music therapy. Studies on behavior observation are crucial for our practical work; however, they only capture what is observable from the *outside* of UWS patients. Neuroscience provides deeper insight into neurological processes and the neurological rehabilitation and has in recent years helped gain a better understanding of the effect of music as a stimulus in the brain. In order to achieve a better understanding of music therapy, it is important to find a more complex approach, combining video analysis, neuroanthropological methods, psycho-vegetative parameters (e.g., heart rate variability) and brain imagery.

Neuroscience helps music therapy gain knowledge about the physiological effects of musical elements, which is useful for the theoretical foundation of music therapy. However, we have to be aware that research on *music therapy* needs a broader approach and interpretation of results than research on music. As the concept of music therapy in Krems, as described above, derives from an anthropological perspective, our approach to research is influenced by a neuroanthropological one (Vogl et al., 2015). Neuroanthropology combines neuroscientific and anthropological research by investigating the interaction between brain, environment and culture. It allows a broader perspective on music therapy by collecting quantitative data as well as qualitative information on the patient's cultural background, environmental influences and the therapeutic relationship between the patient and the therapist. To achieve a careful interpretation of behavioral reactions and imaging results, neuroanthropology encourages a reflective process at any time of a research project and poses profound questions on the meaning of results. PET scans, for example, are important to gain insight into the physiological correlation to music therapy, but results give no answer to the question about the meaning of music therapy. What do neural changes mean for patients with UWS? Does the increase in brain activity show an effect in their behavior? What are the advantages of higher brain activity for these patients? And more generally, what does music therapy really do for them?

We should not forget that adapting to the new situation after the lesion of the brain and coping with UWS can pose an emotional challenge and cause a “reorientation syndrome” (Steinhoff, 2012) for the patient as well as their relatives. To bridge the gap between research and practice, our studies are accompanied by a neuroanthropologist, who focuses on cultural and environmental influences on the brain activity of patients with UWS. Further explanations and first examples of the neuroanthropological approach are published by Vogl et al. (2015).

From an anthropological perspective, the aim of music therapy is to transform the foreign, clinical environment (Umwelt, “around-world”) of patients to their contemporaries (Mitwelt, “with-world”) (Binswanger, 1963; Prinds et al., 2013). By addressing the patient individually and opening up to individual needs and reactions, music therapy is formed not only for the patient, but *with* the patient. Given that the therapeutic relationship and the interaction within music therapy promotes rehabilitation, the therapist presents another unexplored element.

In an interdisciplinary team, we deviate from common patterns of investigation and try to find new ways to examine the effect of music therapy on patients with UWS. As described above, music therapy is more than listening to musical stimuli. Therefore, studying its effect needs to include all its elements. An interdisciplinary approach may help find new methods to get answers. Furthermore, a dialogue is necessary between all people and professions involved in a study: physicians, care team, other therapists, anthropologists, nuclear physicians as well as participants' relatives. Everyone can provide information which is beneficial for a good course of the study.

Summing up, music therapy practice can be advanced by neuroscience opening itself up to individual real-life settings and integrating all elements of music therapy, because its benefit may lie exactly in its complexity. Music therapy is a multisensory, emotional, physical and social approach and therefore involves many neurological functions. If we want to meet the individual needs of the patients, music therapy cannot be standardized. Therefore, it is crucial to have research methods within the frame in which the investigation of individual music therapy takes place. Opening up to this complexity requires new ways of thinking which can be enhanced by an interdisciplinary dialogue. Particularly in music therapy, whose theory and methods are based on the combined knowledge of various disciplines, a dialogue with neuroscience can support the evidence for our practical work and provide insight into deeper processes in our patients. Hence, the dialogue between music therapy and neuroscience is seen as an important, fruitful advantage for both disciplines.

### Conflict of interest statement

The authors declare that the research was conducted in the absence of any commercial or financial relationships that could be construed as a potential conflict of interest.
